# Salivary Histatin 5 Level in Women with Vaginal Candidiasis

**DOI:** 10.1155/2022/5279323

**Published:** 2022-06-27

**Authors:** İrem Şenyuva, Cansu Koca, Funda Karabag Çoban, Özgür Tarhan

**Affiliations:** ^1^Usak Training and Research Hospital, Department of Obstetrics and Gynecology, Usak, Turkey; ^2^University Faculty of Dentistry, Department of Maxillo Facial Surgery, Usak, Turkey; ^3^Usak University, Department of Molecular Biology, Usak, Turkey; ^4^Usak University, Faculty of Engineering, Department of Food Engineering, Usak, Turkey

## Abstract

Histatins (Hsts) are considered a prominent member of antimicrobial peptides rich in histidine, bearing antifungal activity against *Candida* species. Hst5 is the most effective among them. Although Hst5 is not found in the cervicovaginal fluid, it has been detected in the human serum. Saliva acts as a mirror, reflecting the cause and effect relationship between several diseases. We aimed to show the salivary Hst5 levels with vaginal candidiasis. Women in the reproductive age group (18–50 years) were enrolled in the study. Patients and controls were classified based on the presence or absence of vaginal discharge suggestive of candidiasis, respectively. Vaginal and salivary samples were collected from all the women. Vaginal samples were cultured for the growth of *Candida* species. Salivary samples were tested by protein electrophoresis to detect Hst5 levels, and the results were compared between the two groups. A total of 80 women were included in this study. The mean age of women in vaginal candidiasis and control groups was 34.25 ± 8.06 and 36.83 ± 7.29 years, respectively. *Candida* species were isolated from the vaginal samples of the patient group (34 *C*. *albicans*, 6 non-*Candida albicans*) but not from the control group. Hst5 levels in the patient and control group were found to be 0.0571 ± 0.003 ng/mL and 0.0641 ± 0,0031 ng/mL, respectively. Hst5 levels were found to be significantly lower in the vaginal candidiasis group (*p*=0.001). We conclude that decreased salivary Hst5 levels in women are associated with vaginal candidiasis. *Candida* infection is a cause or result of lower salivary Hst5 levels, and it may be an important finding for the etiopathogenesis, diagnosis, and treatment of the disease, but further analysis is needed.

## 1. Introduction

Antimicrobial peptides (AMPs) have a broad spectrum of activity, and they act as the first line of defense in the human body and protect against pathogenic microorganisms [1]. Histatins (Hsts) are considered a prominent member of AMPs rich in histidine, bearing antifungal activity against *Candida* species. Moreover, they have immunomodulator and pro-wound healing effects [2].

Hsts are low molecular peptides found in saliva [3, 4]. Hsts consist of 12 peptides. Hsts 1, 3, and 5 have anti-candidal activity. Hst5 is the most effective, and it is generated by proteolysis of Hst3 [5]. Hsts are coded by HIS1 and HIS2 genes on chromosome 4 with a sequence DSHAKRHHGYKRKFHEKHHSHRGY [4]. Amphipathic nature and cationic residues are associated with antifungal activity [4]. The antifungal effect of Hst5 is shown in a murine-vulvovaginal candidiasis model [6].

Candidiasis is a mucosal infection and aside from oral and systemic candidiasis, it is frequently seen in immunocompetent and healthy women [7]. Some factors like antibiotics, diabetes, and immune deficiency can predispose to this infection [6]. *C*. *albicans* is the most common pathogen responsible for 90% of vaginal candidiasis, and the other non-*albicans Candida* (NAC) species are generally seen in about 50% of the cases [8]. Mucosal inflammation is generally the first symptom, followed by vaginal itching and discomfort, as well as cheese-like vaginal discharge [7].

Several antifungal proteins have been found in the cervicovaginal fluid, but Hsts were not detected among them. However, Hst5 has been detected in the human serum, and Hsts are known to be a part of the human innate immune system [9–12].

Saliva acts as a mirror, reflecting the cause and effect relationship between several diseases. Some autoimmune disorders, as well as viral infections such as Sjögren syndrome, HIV, hepatitis C, and local or systemic candidal infections, can cause salivary gland dysfunction due to autoantibodies and inflammatory cytokines [3, 13–15]. On the other hand, saliva acts as a potent diagnostic tool. Diseases such as breast cancer, diabetes, and their markers such as C-erb2, CA15-3, and A-2-HS-glycoprotein can be detected in saliva samples [3, 16].

Thus, this study is aimed to demonstrate for the first time the salivary Hst5 levels in women with vaginal candidiasis and explore the possible association of salivary Hst5 with vaginal candidiasis. The null hypothesis is that there is no relation between vaginal candidiasis and salivary Hst5 levels.

## 2. Materials and Methods

This study was approved by Usak University Ethical Committee (decision number: 196–01/19.06.2019), and the Number of Clinical Trial (NCT) was NCT05044156. Informed consent form was obtained from all the patients.

Women presenting with vaginal discharge to the Obstetrics and Gynecology department at Usak Training and Research Hospital were evaluated in September–November 2021. This study comprised women 18 to 50 years old who were in the reproductive age group and had vaginal discharge suggestive of candidiasis as the patient group and women with no complaints of vaginal discharge as the control group. Women with diabetes, immune deficiencies, intrauterine devices, pregnancy, virginity, and those in the menopausal stage, as well as women with other types of vaginitis, were excluded from the study [17]. A total of 80 women were enrolled, and vaginal samples were collected in the sterile container for culture from both the patient and control groups. Women were subsequently referred to the Usak University Dental Faculty for an oral examination and saliva sampling. Vaginal samples were cultured for the growth of *C*. *albicans* and NAC. Salivary samples were subjected to protein electrophoresis, and Hst5 levels were determined. The results of the patient and control groups were compared.

### 2.1. Oral Examination and Salivary Sampling

Panoramic radiography and intraoral examination were performed as per the criteria set by the World Health Organization [18]. Saliva samples were taken between 8:30 and 10:30 in the morning, at least one hour after the patients had their breakfast. Unstimulated saliva samples were collected using the oral infusion technique. The patients spit their saliva for 10 min into the centrifuge tubes. It was ensured that the patients do not have any dental pathology (dental caries or periodontal problems) that will affect the content or characteristics of the saliva and have at least 10 teeth. The absence of systemic (Sjögren's syndrome) or local (sialolith) pathological factors that will affect the salivary flow of the patients was also ascertained.

### 2.2. *Candida* Species Isolation

Vaginal samples were inoculated in sterile tubes with 9 cc of Sabouraud Dextrose Broth (SDB) (Oxoid, England) and incubated aerobically for 24 h at 37°C. A 10 *μ*L sample was then taken from each sterile tube and inoculated on Sabouraud Dextrose Agar (SDA) (Oxoid, England) with chloramphenicol and gentamicin. Petri dishes were incubated at 37°C in an aerobic environment for 24–72 h, and growth from at least 5 colonies was analyzed. These colonies were examined macroscopically, followed by Gram stain, for microscopic examination. *Candida* species and other organisms were identified in the isolates [19].

### 2.3. Salivary Sample Protein Electrophoresis

Saliva protein electrophoresis was performed on 35 salivary samples from patients and controls using lyophilized Hst5 (826319, MBS Inc, San Diego, USA). Protein fractions in saliva were analyzed by sodium dodecyl sulfate-polyacrylamide gel electrophoresis (SDS-PAGE) according to the Laemmli protocol [20].

### 2.4. Salivary Sample Hst5 Level Measurement

The ELISA technique was used to analyze salivary Hst5 samples [10]. The Human Histatin 5 ELISA Kit (E1444 Hu, BT Lab, Shanghai, China) was used.

Sample size calculation was performed with a computer application G^*∗*^ Power software (latest ver. 3.1.9.7), and the calculation was performed considering the results of a previous study, reporting a mean Hst5 of 18.64 *μ*g/ml for the test group and 24.94 *μ*g/ml for a control group [21]. If the effect size is 0.8 and the sample size is 80, the possibility of the study identifying a difference at a two-sided 0.05 significance level is 90% (power 0.9). NCSS (Number Cruncher Statistical System) 2007 (Kaysville, Utah, USA) was used for statistical analyses. The data were analyzed using descriptive statistics (mean, standard deviation, median, frequency, rate, minimum, and maximum), and the Shapiro–Wilk test was used to assess data distribution. The Kruskal–Wallis test was used for quantitative analysis to compare three or more groups, while the Mann–Whitney *U* test was used to compare two groups. For comparing quantitative data, Spearman's correlation analyses were employed. The significance levels of *p*=0.01 and *p*=0.05 were applied.

## 3. Results

The mean age of women was found to be 34.25 ± 8.06 years and 36.83 ± 7.29 years in the vaginal candidiasis and control groups, respectively (*p*=0.135). The body mass index (BMI) of the women in vaginal candidiasis and control groups was 26.56 ± 5.66 and 26.44 ± 5.09, respectively (*p*=0.706).

**Figure 1:**
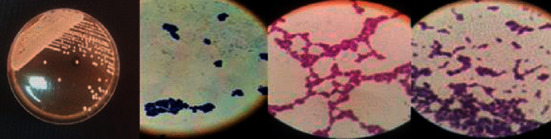
Colonization of *Candida* species in SDA and gram strain microscopic view (SDA: Sabouraud Dextrose Agar).

*Candida* species isolated from the patient group included *C*. *albicans* (34) and NAC (6). Samples from none of the 40 women in the control group revealed growth of *Candida* species. Colony characteristics of *Candida* species on the SDA and microscopic view of gram stain are shown in Figure 1.

Macroscopic findings of women's saliva sampling were observed to be dark, viscous, and foamy in the vaginal candidiasis group; in the control group, saliva samples were juicy and clear.

**Figure 2:**
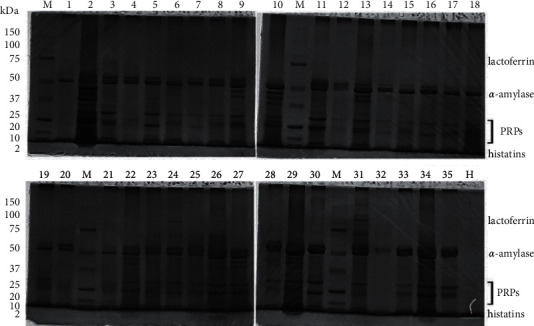
SDS-PAGE profiles of salivary samples. Lanes represented by 1 to 35 indicate patient and control. M represents protein marker (Biorad 1610377). H represents histatin 5 standard (MyBioSource MBS826319).

Hst5 protein bands were detected in salivary samples using SDS-PAGE, as demonstrated in Figure 2.

Hst5 levels in women with vaginal candidiasis and controls were found to be 0.057 ± 0.003 ng/ml and 0.0641 ± 0.003 ng/ml, respectively, and a lower Hst5 level was found to be significantly associated with vaginal candidiasis (*p*=0.001) (Table 1).

## 4. Discussion

In present study, we detected lower salivary Hst5 level in women with vaginal candidiasis.

Colonization of the yeast form of *Candida* species in the vaginal epithelium is the first step in vaginal candidiasis [7]. Then, they convert to hypha form via hypha-associated virulence factors. This stimulates NOD, LRR, and pyrin domain-containing protein 3 (NLRP3) inflammasome signaling, causing inflammatory cytokines and chemokines to migrate to the site of infection, followed by polymorphonuclear leukocyte invasion in the vaginal epithelial lamina propria [22]. Hst5 is an antifungal agent with fungicidal activity against *C*. *albicans* and NAC during all these stages of pathogenesis [12]. Hst5 exerts biological effects through binding directly to glucans in fungal cell walls and heat shock proteins, it enters the fungal cells through the fungal polyamine transporters Dur 3 and Dur 31 via energy-dependent translocation, and Ssa1-Ssa2 act as a binding protein in the cell wall [23]. All of these processes are dependent on Hst5 levels, and higher physiologic Hst5 levels have a more profound effect [6]. Hst5 causes potassium release, cell volume impairment, mitochondrial dysfunction via ATP efflux, and production of reactive oxygen species (ROS) in the intracellular medium [12]. Hst5 binds to the metal as well [24]. Hst5 stability against protease is improved by the presence of iron, copper, zinc, and nickel [25]. *In vitro* studies have revealed that Hst5 increases fungal hydrolysis by binding to host zinc [25, 26]. *In vitro* studies have also shown that Hst5 has a synergistic lethal impact on *Candida* species when combined with AMPs such lactoferrin and defensin, which are found in the female vaginal tract [27–29]. Potent antifungal activity seen in Hst5 has not been observed in any other AMPs in humans since it is associated with the R-K-F-H-E-K-H-HS-H-R sequence and is included within the Hst functional domain, making Hst unique [30, 31]. Unfortunately, Hst5 does not occur in the cervicovaginal fluid AMPs, but it is found in the blood [10, 11]. Given that decreased Hst5 level could lead to vaginal candidiasis, the null hypothesis could be rejected because lower Hst5 levels may affect the vaginal area by inhibiting *Candida* colonization through the bloodstream. In such a case, the cause of the low salivary Hst5 level could be explained by genetic predisposition. Genetic polymorphism in innate immunity genes such as mannose-binding lectin (MBL), dectin-1 stop-codon, interleukin 4 (IL-4), and NLRP3 was found to be related to vulvovaginal candidiasis among women with no predisposing factors [32]. Current literature has reported that genetic variability, impaired transcriptional control, expression of Hst protein, HIS mutation, and Hst3-2 protein variations are linked [33, 34]. Further research is needed to determine the link between HIS gene mutation and functional outcomes.

Considering that vaginal candidiasis may lead to decreased salivary Hst5 level, the null hypothesis could be rejected. *Candida*, on the other hand, has developed a way to resist the action of Hst5 [35]. Hst5 breaks down candidal virulence factors such as secreted aspartyl proteases (Sap) and membrane-bound-signaling mucin (Msb2), and this mechanism is linked to Cek1 mitogen-activated protein kinase (MAPK) pathways [12, 36]. The Flu1efflux pump, which is implicated in multidrug resistance, has been discovered to transport Hst5 out of candidal cells [37, 38]. Furthermore, *Candida* species may reduce salivary gland secretion in addition to degrading Hst5. In viral diseases like HIV and hepatitis C, antiviral antibodies have been detected in the saliva, which deranges the salivary gland function [39, 40]. *Candida* species, similar to these infections, may change salivary protein levels, resulting in a low level of Hst5.

We also explored the possibility of oral candidiasis in women with vaginal candidiasis and lower Hst5 levels. Low Hst5 levels have been found in immunosuppressed patients, and salivary Hst5 levels have been shown to promote oral infections in such cases [4]. In the present study, we discovered that all of the women were immunocompetent and had good oral health. Other antifungal salivary proteins, including LL-37, defensin, and calprotectin, may have a protective role against fungal infection [41, 42]. Despite a low Hst5 level in the saliva, oral candidiasis was not observed in our study.

Vulvovaginal candidiasis deteriorates the quality of life, mental health, and sexuality of women [43]. It has been reported that 70–75% of women experience vaginal candidiasis in their lives [33]. Azole group of the antifungal agent is commonly used for the treatment of *Candida* infections, although drug resistance may be seen. This necessitates the development of alternative therapeutic options [44]. Some of these include boric acid, probiotics, and immunotherapeutic vaccines [44, 45]. Liano et al. [6] reported that Hst5 is a powerful antifungal protein in a murine vaginal candidiasis model. Hst5 is a potent peptide because their proteolytic fragments called P-113 retain their anti-candidal activity and do not easily break, but they can be broken down by environmental factors, especially in in vivo conditions [25]. It has also been established that there is a particular Hst5 binding domain on the *C*. *albicans* surface, which is crucial for selectivity and lack of toxicity to the human host cell [46]. If the stability of Hst5 can be enhanced somehow, such as by metal bindings and amino acid changes, it could be a promising option for candidal treatment [25].

### 4.1. Limitations of the Study

Currently, there are many in vivo and in vitro studies about the antifungal activity of Hst5 in the literature; however, clinical studies are lacking. We demonstrated as preliminary salivary Hst5 levels in vaginal candidiasis. We identified *C*. *albicans* and NAC with the help of gram strain and could not specify species. Further research regarding the link between salivary Hst5 levels and *Candida* types in women with vaginal candidiasis needs to be conducted. We did not, on the other hand, investigate in oral and vaginal microbiota or probiotics. Oral probiotics modulate immunity by altering the human microbiota, and the oral microbiota changes the profile of oral AMPs [47–50]. The bacterial microbiota on the mucosal layer of the vagina maintains acidic pH, releases antifungal peptides, and provides physiological regulation against dysbiosis [50]. Lactobacillus production of lactic acid has been shown to suppress *C*. *albicans* hyphal development; conversely, elevated pH levels can contribute to vaginal candidiasis [7]. Hst5 is a cationic peptide, and acidic pH values increase Hst5's cationic charge, intracellular uptake, and killing effect [51]. Oral probiotics could have a positive effect on saliva Hst5 levels, altering vaginal pH and enhancing the effects of Hst5. The relationship between salivary Hst5 levels, microbiota, and probiotics in women with vaginal candidiasis needs to be investigated.

## 5. Conclusion

*Candida* species is a causative agent for vaginal candidiasis in many women throughout their lives. *Candida* infection may be a cause or effect of lower salivary Hst5 levels. It could be an important finding for the etiopathogenesis, diagnosis, and treatment of the disease. Further research is required to confirm the findings. Salivary tests may be a simple and easy diagnostic tool for detecting Hst5 levels in the future, and Hst5 replacement may be a rational choice for treating vaginal candidiasis.

## Figures and Tables

**Table 1:** Hst5 levels of study groups.

	Hst5 level (ng/ml)		*P*
	Min-max	Mean ± sd	
Vaginal candidiasis (*n* = 40)	0.05–0.06	0.057 ± 0.003	0.001
Control (*n* = 40)	0.06–0.07	0.0641 ± 0.0031	

## Data Availability

The data used to support the findings of this study are included within the article.
